# ERK inhibition promotes neuroectodermal precursor commitment by blocking self-renewal and primitive streak formation of the epiblast

**DOI:** 10.1186/s13287-017-0750-8

**Published:** 2018-01-05

**Authors:** Yang Yu, Xiaoxiao Wang, Xiaoxin Zhang, Yanhua Zhai, Xukun Lu, Haixia Ma, Kai Zhu, Tongbiao Zhao, Jianwei Jiao, Zhen-Ao Zhao, Lei Li

**Affiliations:** 1 State Key Laboratory of Stem Cell and Reproductive Biology, Institute of Zoology, Chinese Academy of Sciences, 1 Beichen West Road, Chaoyang District, Beijing, 100101 China; 2 University of Chinese Academy of Sciences, Beijing, 100049 China; 3 Department of Cardiovascular Surgery of the First Affiliated Hospital & Institute for Cardiovascular Science, Soochow University, 708 Renmin Rd, Suzhou, Jiangsu 215007 China

**Keywords:** Extracellular signal-regulated protein kinase, Neural differentiation, PD0325901, Epiblast stem cells, Embryonic stem cells

## Abstract

**Background:**

Pluripotent stem cells hold great promise for regenerative medicine. However, before clinical application, reproducible protocols for pluripotent stem cell differentiation should be established. Extracellular signal-regulated protein kinase (ERK) signaling plays a central role for the self-renewal of epiblast stem cells (EpiSCs), but its role for subsequent germ layer differentiation is still ambiguous. We proposed that ERK could modulate differentiation of the epiblast.

**Methods:**

PD0325901 was used to inhibit ERK activation during the differentiation of embryonic stem cells and EpiSCs. Immunofluorescence, western blot analysis, real-time PCR and flow cytometry were used to detect germ layer markers and pathway activation.

**Results:**

We demonstrate that the ERK phosphorylation level is lower in neuroectoderm of mouse E7.5 embryos than that in the primitive streak. ERK inhibition results in neural lineage commitment of epiblast. Mechanistically, PD0325901 abrogates the expression of primitive streak markers by β-catenin retention in the cytoplasm, and inhibits the expression of OCT4 and NANOG during EpiSC differentiation. Thus, EpiSCs differentiate into neuroectodermal lineage efficiently under PD0325901 treatment. These results suggest that neuroectoderm differentiation does not require extrinsic signals, supporting the default differentiation of neural lineage.

**Conclusions:**

We report that a single ERK inhibitor, PD0325901, can specify epiblasts and EpiSCs into neural-like cells, providing an efficient strategy for neural differentiation.

**Electronic supplementary material:**

The online version of this article (doi:10.1186/s13287-017-0750-8) contains supplementary material, which is available to authorized users.

## Background

Mouse embryonic stem cells (mESCs) are established from the inner cell mass of preimplantation embryos at embryonic day 3.5 (E3.5) [1, 2]. Mouse epiblast stem cells (mEpiSCs) are derived from the postimplantation epiblast from E5.5 to E7.5 and share defining features with human embryonic stem cells (hESCs) rather than mESCs [3–5]. Although there are marked differences between mESCs and mEpiSCs, both cell lines have the ability to give rise to all three embryonic germ layers in vivo and in vitro, holding great promise for regenerative medicine. However, the development of reproducible and highly efficient protocols for ESC differentiation is still needed for the clinical application of these cells [6].

Gastrulation is marked by formation of the primitive streak (PS); uncommitted epiblast cells traverse through the PS and differentiate into either the mesoderm or definitive endoderm, whereas anterior epiblast cells that do not enter the PS differentiate into the neuroectoderm [7]. The NODAL/SMAD2 signaling pathway has been shown to promote postimplantation development, and deletion of this pathway results in the absence of PS formation [8, 9]. Furthermore, NODAL induces BMP4 expression in the extraembryonic ectoderm, and BMP4 signaling upregulates WNT3, which in turn maintains NODAL expression through regulating its enhancer directly [10–12]. Additionally, WNT/β-catenin signaling also controls PS differentiation through interaction with the SMAD signaling pathway [13]. Above all, gastrulation is finely regulated through the coordinated activities of several signaling pathways, including NODAL, WNT, and BMP.

FGF/ERK signaling is a highly conserved pathway regulating cell proliferation, differentiation, and apoptosis, and it is involved in a multitude of developmental processes [14]. Although *Erk1*-deficient mice are viable and fertile [15], mouse embryos absent of *Erk2* fail to form the mesoderm, and the embryos are lethal at E6.5 [16]. In addition, the depletion of mouse *Fgf8* or its receptor, *Fgfr1*, leads to severe gastrulation defects [17, 18]. Thus, FGF/ERK signaling is essential for gastrulation during mouse early development. The role of FGF/ERK signaling in stem cell differentiation and lineage commitment is not understood fully. Meanwhile, the mechanism of FGF/ERK signal is still largely unknown.

In the present study, we found that ERK activity in the neuroectoderm of E7.5 embryos was much lower than that in the primitive streak, supporting a role for ERK inhibition in neural differentiation. Temporal FGF/ERK signal inhibition directed mouse E5.75 epiblast cells and mEpiSCs into neural fate efficiently, and abrogated the PS induction by Activin A and WNT pathways. Moreover, we found that inhibition of ERK contributed to differentiation of the neural lineage through preventing PS differentiation by modulating β-catenin retention in the cytoplasm, and inhibiting the self-renewal of epiblast cells by the downregulation of OCT4.

## Methods

### Mice

ROSA^mT/mG^ (J007676) [19] and Nes-Cre (J003771) [20] mice were obtained from NBRI (Nanjing Biomedical Research Institute of Nanjing University; http://www.nbri-nju.com/en-us/). Commercial ICR, C57Bl/6, and 129S2/SvPasCrlVr mice were purchased from Vital River Laboratories. These strains were maintained under SPF conditions in the animal facilities at Institute of Zoology, Chinese Academy of Sciences. Animal experiments were conducted in accordance with the guidelines of the Institutional Animal Care and Use Committee at Institute of Zoology, Chinese Academy of Sciences.

### Mouse ESC and EpiSC culture

CMTI-1 (Millipore) and 46C (Sox1-GFP) ESCs were maintained in DMEM containing 15% FCS, 1000 units/ml leukemia inhibitory factor (LIF, Esgro, catalog number ESG1107; Millipore), 2 mM Glutamax, 100 units/ml penicillin/streptomycin, 0.1 mM nonessential amino acids, 0.1 mM 2-mercaptoethanol, and 1 mM sodium pyruvate [21]. Naïve mESCs were maintained on gelatin-coated dishes in 2i/LIF conditions defined as N2B27 medium supplied with 1000 units/ml LIF, 1 μM PD0325901 (LC Laboratories), and 3 μM CHIR99021 (LC Laboratories) [22].

EpiSCs were derived from mouse E5.75 embryos (129S2 × C57Bl/6, F1) as reported previously [3, 4]. EpiSC culture medium consisted of 15% KSR (Invitrogen), 10 ng/ml Activin A (Peprotech), 5 ng/ml FGF2 (Peprotech), and 2 μM XAV939 (Sigma-Aldrich) in N2B27. EpiSCs were passaged every 2 days with 1.5 mg/ml collagenase IV (Invitrogen) as small clumps on Mitomycin C inactivated MEFs or Matrigel-coated dishes (BD Biosciences). Sox1-GFP EpiSCs were derived from 46C cells by culturing in EpiSCs medium for 7 passages.

### Separation of postimplantation germ layers

Epiblasts were isolated from mouse E5.5–6.5 embryos. The primitive streak and neuroectoderm were isolated from E7.5 embryos in DMEM medium containing 10% FBS and 25 mM HEPES. Reichert’s membrane was removed with fine forceps, and the embryonic region was separated by a glass scalpel. After two washes in DPBS, the tissues were incubated in a digestion solution at 4 °C for 8–12 minutes. The digestion solution consisted of 0.5% trypsin and 2.5% pancreatin in Ca^2+^/Mg^2+^-free Tyrode Ringer’s Saline (pH 7.6–7.7). Germ layers were separated with Pasteur pipettes and glass needles. For western blot analysis, the tissues were rinsed twice in DPBS, and transferred to lysis buffer directly.

### Stem cell differentiation

Mouse EpiSCs and E5.75 epiblasts were digested with 0.25% trypsin and neutralized with ESC medium. The cells were then pipetted into small clumps and incubated in 2i/LIF medium on MEF feeder. Round colonies were observed after a 3-day culture. The colonies were digested into single cells and replated in 2i/LIF medium.

ESC neuroectodermal precursor commitment in the monolayer was performed as reported previously [21]. In total, 4 × 10^4^ ESCs were replated on 0.1% gelatin-coated 35-mm dishes in N2B27 medium for 5 days. The medium was changed every 2 days. Then, 1 μM MEK inhibitor PD0325901 was added at different time points to promote neuroectodermal precursor differentiation.

For EpiSC differentiation, cell clumps were seeded on Matrigel-coated dishes. After 1 day of culture, primitive streak differentiation was induced in N2B27 by adding 10 ng/ml Activin A or 3 μM CHIR99021, and neuroectoderm differentiation was induced by 2 μM SB431542 (Axon).

### Western blot analysis

Normalized protein samples were separated in 10% SDS-PAGE gel and transferred onto PVDF membrane. The membranes were blocked with 5% nonfat milk and incubated with diluted primary antibodies at 4 °C overnight. The incubation of secondary antibodies (1:3000; Jackson ImmunoResearch) was performed at room temperature for 1 hour. The signals were developed with Pierce ECL Substrate in Bio-RAD ChemiDocTMXRs + (Bio-Rad Laboratories) with Quantity One software. The primary antibodies included: anti-phospho (Thr202/Tyr204)-ERK1/2 (1:2000, #4370; CST), anti-ERK1/2 (1:1000, #4695; CST), anti-phospho (Ser465/467)-SMAD2 (1:1000, #3108; CST), anti-phospho (Ser245/250/255)-SMAD2 (1:1000, #3104; CST), anti-SMAD2 (1:1000, #5339; CST), anti-SMAD2/3(1:1000, #8685; CST), anti-SMAD4 (1:1000, #9515; CST), anti-β-catenin (1:1000, ab6302; Abcam), anti-non-phospho (Active) β-catenin (1:1000, #8814; CST), anti-OCT4 (1:500, sc-8628; Santa Cruz), anti-FOXA2/HNF3β (1:1000, #8186S; CST), anti-brachyury (T) (1:500, sc-17743; Santa Cruz), anti-SOX2 (1:1000, AB5603; Millipore), anti-NANOG (1:1000, ab80892; Abcam), anti-E-cadherin (1:1000, #3195; CST), anti-N-cadherin ( 1:500, ab76057; Abcam), anti-Lamin A/C (1:1000, #4777; CST), anti-GAPDH (1:1000, DKM9002T; Sungene Biotech), anti-β-actin (1:1000, A2228; Sigma-Aldrich), and anti-α-tubulin (1:1000, #2144; CST). Quantitative analysis of blots was performed using ImageJ software.

### Real-time PCR

Total RNA was isolated with an RNeasy mini kit (Qiagen) and the concentrations were measured with NanoDrop 2000 (Thermo Fisher). cDNA was reverse-transcribed from 500 ng RNA using the PrimeScript RT Reagent Kit (TaKaRa). Real-time PCR was performed using a SYBR Premix Ex Taq kit (TaKaRa) on a LightCycler 480 II (Roche). The data were generated from at least three independent biological samples. *Hprt* was used as an internal reference gene for normalization, and the 2^–ΔΔCt^ method was used for data analysis [23]. Primer sequences for real-time PCR are presented in Additional file 1: Table S1.

### Immunofluorescence and flow cytometry analysis

Cells were fixed with 4% PFA for 30 minutes and incubated in 1% Triton-X-100 for 15 minutes to permeate the cell membrane. Nonspecific binding was blocked with 1% BSA at room temperature for 1 hour. Proteins were detected with specific primary antibodies at 4 °C overnight. Primary antibodies were as follows: anti-NESTIN (1:100, MAB353; Millipore), anti-TuJ 1 (1:200, T2200; Sigma-Aldrich), anti-OCT4 (1:200, sc-8628; Santa Cruz), anti-NANOG (1:200, ab80892; Abcam), anti-phospho (Ser465/467)-SMAD2 (1:200, #3108; CST), and PAX6 (1:200, AB_528427; DSHB). After three washes with PBS, cells were incubated with corresponding secondary antibodies (1:1000; Jackson ImmunoResearch) for 1 hour. DNA was counterstained with Hoechst33342 (Invitrogen) for 5 minutes at room temperature. Immunofluorescent images were obtained on an Axioplan Zeiss microscope (LSM 780; Carl Zeiss). Quantitative analysis of immunofluorescent staining was performed using ImageJ software when the immunofluorescent images were obtained at the same exposure parameters.

For FACS analysis, cells were digested into single cells, followed by two washes in DPBS. The cells were then filtered through a 35-μm cell strainer cap (Falcon™ Cell Strainers, 352235). Sox1-GFP cells were sorted and counted by flow cytometry. Analysis was performed on a FACS-Canto flow cytometer (Beckman Coulter MoFlo™ XDP).

### Statistical analyses

Statistics were calculated using SPSS 18.0 software. The data were subjected to Student’s *t* test or one-way analysis of variance (ANOVA) for significance analysis (*p* < 0.05). The data were from at least three independent biological samples and expressed as the mean ± SEM.

## Results

### Epiblast cells commit to the neural lineage in 2i/LIF medium

**Fig. 1:**
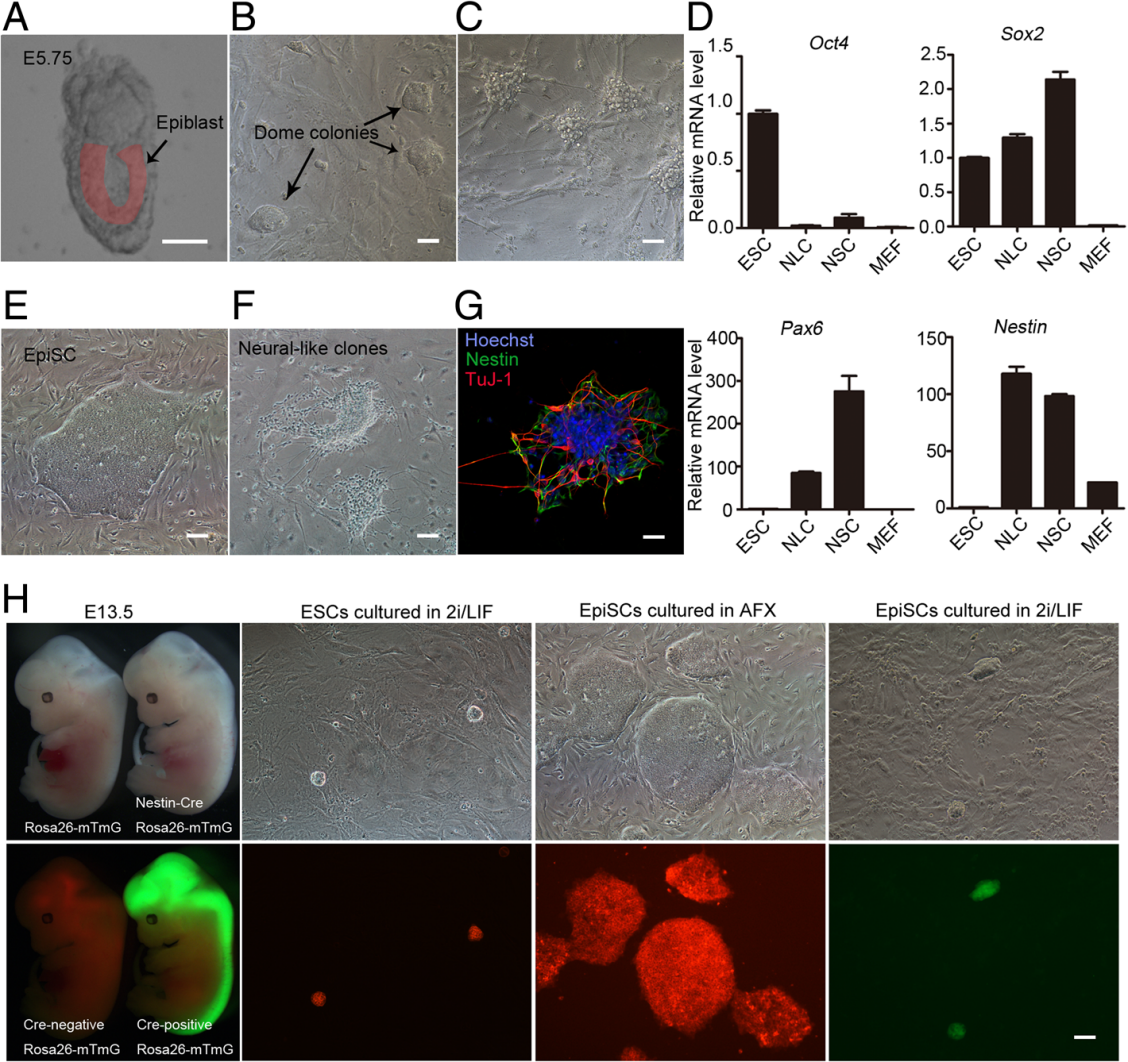
Epiblast cells were committed to neural lineage in 2i/LIF culture condition. **a** Epiblasts isolated from mouse E5.75 embryos. **b** Small domed colonies appeared after culturing epiblast cell clumps on MEF feeder in 2i/LIF medium for 3 days. **c** All clones exhibited neural-like morphology after two passages in 2i/LIF medium. **d** Real-time PCR showed the mRNA expression pattern of neural-like clones (NLC) was similar to neural stem cells (NSC) other than ESCs. Pluripotent markers, *Oct4* and *Sox2*; neuroectoderm markers, *Sox2* and *Pax6*; neural stem cell marker, *Nestin*. RNA collected on day 3 after replating the domed colonies. **e** Morphology of EpiSCs isolated from mouse E5.75 epiblasts. **f** NLC emerged by passaging EpiSCs in 2i/LIF medium twice. **g** NLC expressed the neural stem cell marker NESTIN (green) and the neuron marker TuJ-1 (red). Nuclei counterstained by Hoechst 33342 (blue). **h** Embryos were obtained from ROSA^mT/mG^ × Nes-cre mouse strains. ROSA^mT/mG^ × Nes-cre E13.5 embryos expressed GFP in the neural system. Only domed ESC clones cultured in 2i/LIF medium and flattened EpiSCs maintained in AFX medium (10 ng/ml Activin A, 5 ng/ml FGF2, and 2 μM XAV939) expressed red tomato; however, domed colonies expressing GFP were observed after EpiSCs were cultured in 2i/LIF for 3 days, indicating CRE activity driven by the *Nestin* promoter. Bar, 100 μm. E embryonic day, ESC embryonic stem cell, MEF mouse embryonic fibroblasts, EpiSC epiblast stem cell, OCT4 octamer-binding transcription factor 4, *Pax6* paired box 6, SOX2 sex determining region Y-box 2

2i/LIF medium, containing MEK inhibitor PD0325901 (PD), GSK3 inhibitor CHIR99021 (CHIR), and LIF, is generally used for mouse ESC culture. Mouse EpiSCs can be reverted into mESCs in 2i/LIF medium at low efficiency [24, 25]. To examine whether mouse ESCs could be established from mouse E5.75 embryos in 2i/LIF medium, we isolated E5.75 epiblasts (Fig. 1a), digested the tissues into small cell clumps, and cultured them in 2i/LIF medium for 3 days. Some small domed colonies that were similar to mESC clones appeared under these conditions (Fig. 1b). These colonies were then digested into single cells and further cultured in 2i/LIF medium. Interestingly, no ESC-like clones appeared, but neural-like clones emerged after 3 days of culture (Fig. 1c). To confirm their neural fate, these cells were examined by real-time PCR with specific primers for *Oct4*, *Sox2*, *Pax6*, and *Nestin*. Compared with mESCs, the neural-like cells exhibited lower expression of *Oct4* and higher neural marker expression, including *Pax6*, *Sox2*, and *Nestin* (Fig. 1d). These results showed that most of the epiblast cells from E5.75 mouse embryos differentiated into neural-like cells, but not ESCs, when they were cultured in 2i/LIF medium.

We then investigated whether mEpiSCs could differentiate into neural-like cells in 2i/LIF medium. To do this, we established mouse EpiSCs from E5.75 mouse epiblast as reported previously [3, 4, 26]. Typical EpiSC morphology was observed (Fig. 1e), similar to previous reports [3–5, 26]. The mouse EpiSCs were then transferred into 2i/LIF medium and further cultured under this condition. Consistent with the earlier observations, mouse EpiSCs differentiated into neural-like clones after two passages in 2i/LIF culture medium instead of reverting to ESC clones (Fig. 1f). The neural differentiation of EpiSCs in 2i/LIF was further verified by immunofluorescence staining with Nestin and TuJ-1 antibodies (Fig. 1 g). These data suggested that mouse EpiSCs differentiate into neural lineage cells, rather than ESCs, in 2i/LIF culture condition.

To further confirm the differentiation of mouse EpiSCs into neural-like cells, we isolated mouse ESCs and EpiSCs from the mouse line by mating ROSA^mT/mG^ mice with Nes-Cre (neural cell lineage) mice. In the ROSA^mT/mG^ mouse line, the membrane-targeted tandem dimer tomato (mT) is expressed prior to Cre-mediated excision, and membrane-targeted green fluorescent protein (mG) is expressed after Cre excision [19]. The conversion of tomato into GFP driven by Nes-Cre was used to trace neuroectodermal precursor commitment of ESCs and EpiSCs (Fig. 1 h). Mouse ESCs cultured in 2i/LIF and undifferentiated EpiSCs expressed mT (red); however, GFP-positive clones were observed when EpiSCs were cultured in 2i/LIF medium (Fig. 1 h). Thus, both in-vivo epiblast cells and in-vitro EpiSCs were committed into neuroectodermal precursors under 2i/LIF medium.

### PD0325901 promotes neuroectodermal precursor formation of EpiSCs

**Fig. 2:**
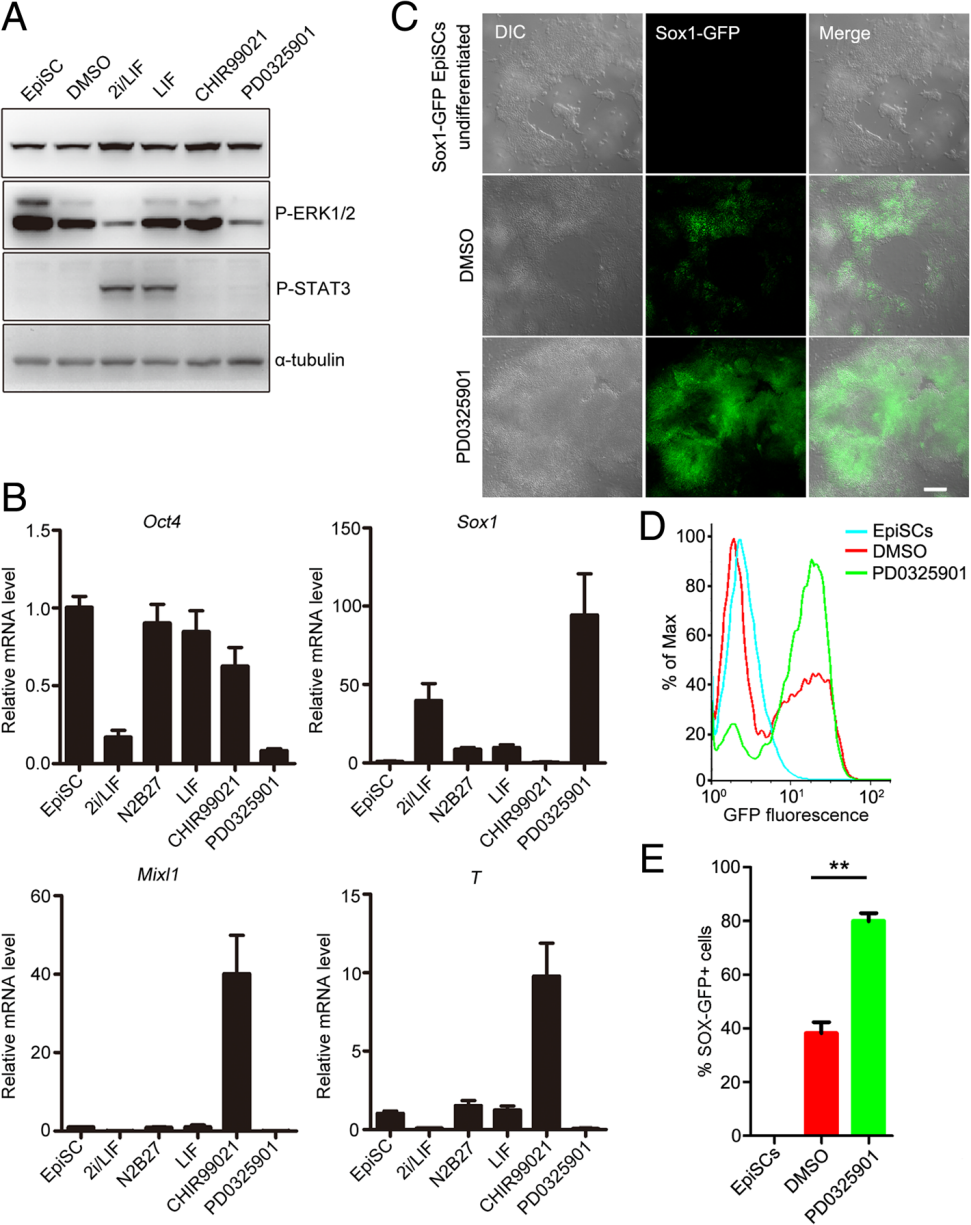
PD0325901 in 2i/LIF was responsible for neural differentiation of EpiSCs. **a** Activities of signal pathways were detected by western blot analysis. β-catenin and STAT3 were activated by 3 μM CHIR99021 and 1000 U/ml LIF, respectively; Phospho-ERK1/2 was inhibited by 1 μM PD0325901. **b** Real-time PCR results showed that PD0325901 in 2i/LIF induced neuroectoderm marker *Sox1*, and inhibited pluripotent marker *Oct4* and primitive streak markers *T* and *Mixl1*. **c** PD0325901 treatment for 24 hours dramatically increased the proportion of GFP-positive cells, when Sox1-GFP EpiSCs were cultured in N2B27 medium. GFP-positive cells were detected by fluorescence microscopy. Bar, 100 μm. **d**, **e** Sox1-GFP EpiSCs were cultured in N2B27 containing 0.01% DMSO or 1 μM PD0325901 for 24 hours. GFP-positive cells were analyzed by FACS. **p < 0.01. DMSO dimethyl sulfoxide, EpiSC epiblast stem cell, ERK extracellular signal-regulated protein kinase, GFP green fluorescent protein, DIC differential interference contrast, LIF leukemia inhibitory factor, *Mixl1* mix paired-like homeobox, *Oct4* octamer-binding transcription factor 4, *T* brachyury, *Sox1* sex determining region Y-box 1, STAT3 signal transducer and activator of transcription 3

To examine which components in 2i/LIF medium contributed to the differentiation of the neural lineage, mouse EpiSCs were treated with PD0325901, CHIR99021, or LIF in N2B27 medium. Consistent with their roles in mouse ESCs [22], CHIR99021 and LIF activated β-catenin and STAT3, respectively, and PD0325901 inhibited ERK1/2 phosphorylation (Fig. 2a, Additional file 2: Figure S1). After one day of treatment, PD0325901 dramatically induced neural marker *Sox1* expression and inhibited pluripotent marker *Oct4* expression, and CHIR99021 markedly induced the expression of primitive streak markers (*Mixl1* and *T*) (Fig. 2b). Compared to the control, LIF had no significant effect on cell differentiation of mEpiSCs (Fig. 2b). These data showed that PD0325901 was the functional component in 2i/LIF medium, suggesting that inhibition of ERK1/2 activity by PD0325901 promotes the formation of neuroectodermal precursors during mouse EpiSC differentiation.

To further investigate the role of ERK1/2 inhibition during EpiSC differentiation into neuroectodermal precursors, mouse Sox1-GFP EpiSCs were established from 46C mESCs by culturing in EpiSC culture medium for seven passages [21, 27]. Then, mouse Sox1-GFP EpiSCs were used to trace neural lineage formation during EpiSC differentiation. Mouse Sox1-GFP EpiSCs were negative for GFP (Fig. 2c–e), but 38.2% of cells expressed GFP when they were differentiated spontaneously in N2B27 medium for 24 hours (Fig. 2c–e). PD0325901 treatment significantly promoted the proportion of GFP-positive cells to 79.9% (Fig. 2c–e). Thus, inhibition of ERK1/2 activity by PD0325901 significantly promotes the commitment of neuroectodermal precursors during mouse EpiSC differentiation.

### Inhibition of ERK prevents primitive streak formation and self-renewal of EpiSCs

**Fig. 3:**
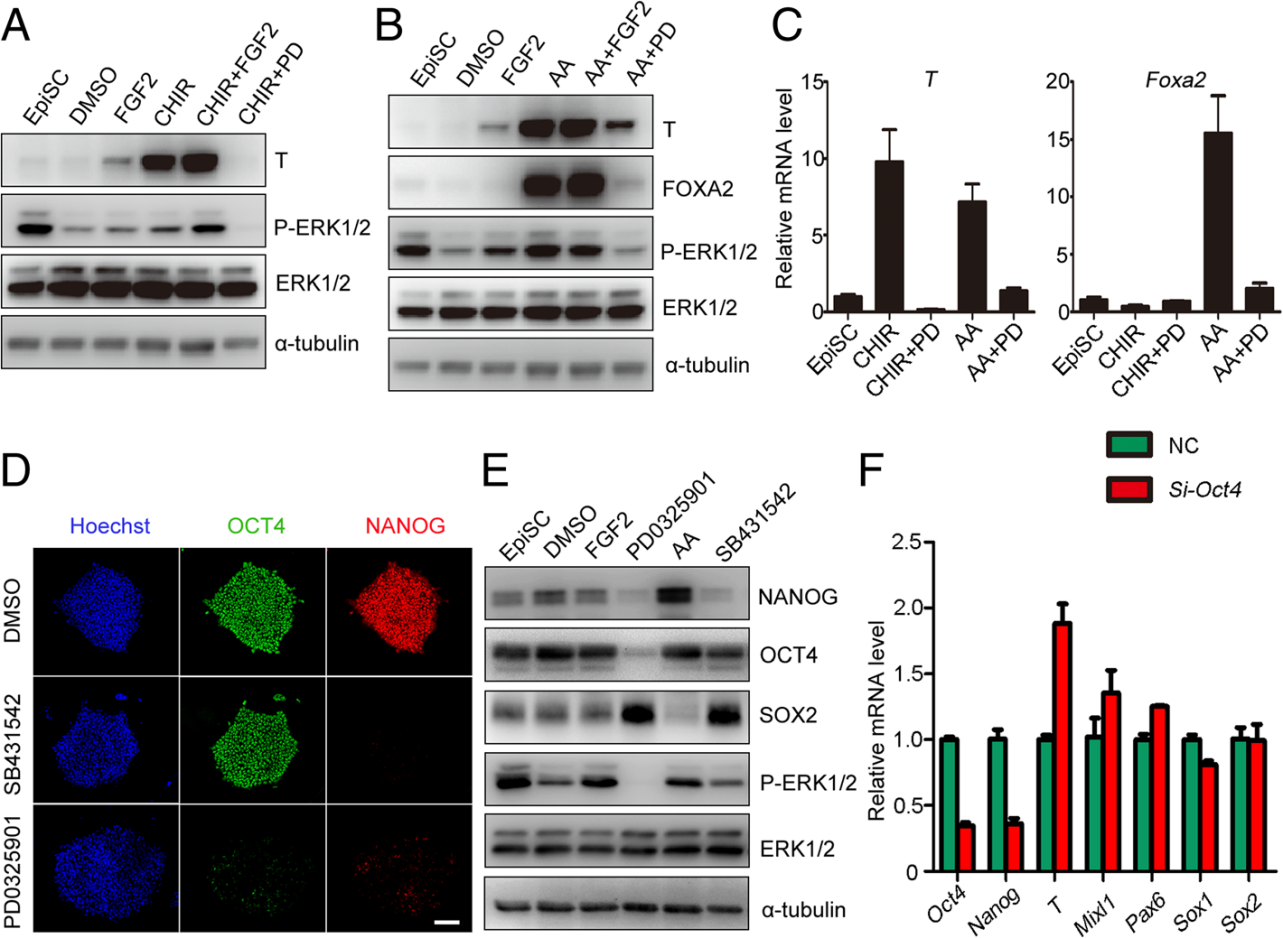
PD0325901 prevented formation of the primitive streak and inhibits EpiSC self-renewal. **a** Western blot analysis showed PD0325901 (PD, 1 μM) prevented expression of PS marker T in the presence of CHIR99021 (CHIR, 3 μM) in EpiSCs cultured in N2B27 for 24 hours. **b** PD0325901 prevented expression of PS marker T and endoderm marker FOXA2 in the presence of Activin A (10 ng/ml) in EpiSCs cultured in N2B27 for 24 hours. **c** Real-time PCR showed that PD0325901 inhibited differentiation of PS even in the presence of Activin A or CHIR99021. **d** Immunostaining showed that PD0325901 inhibited both OCT4 and NANOG expression, whereas SB431542 (2 μM) only inhibited NANOG expression in EpiSCs differentiated in N2B27 for 24 hours. Bar, 100 μm. **e** PD0325901 inhibited both OCT4 and NANOG expression detected by western blot analysis. **f** *Oct4* knockdown promoted PS differentiation in EpiSCs cultured in N2B27 medium for 24 hours. Pluripotent markers, *Oct4* and *Nanog*; primitive streak markers, *T* and *Mixl1*; endoderm marker, *Foxa2*; neuroectoderm markers, *Sox1, Sox2* and *Pax6.* AA Activin A, NC negative control, DMSO dimethyl sulfoxide, EpiSC epiblast stem cell, FGF fibroblast growth factor, T brachyury, ERK extracellular signal-regulated protein kinase, OCT4 octamer-binding transcription factor 4, FOXA2 forkhead box protein A2, SOX2 sex determining region Y-box 2, *Mixl1* mix paired-like homeobox, *Pax6* paired box 6

The specification of neuroectodermal precursors can be inhibited by WNT, and Activin/Nodal signaling pathways which are important for the formation of the primitive streak during mouse early embryonic development [28–31]. Thus, PD0325901 might improve neuroectodermal precursor differentiation of mouse EpiSCs through blocking the formation of the primitive streak. To test this hypothesis, we treated mouse EpiSCs with FGF2, CHIR99021, Activin A, PD0325901, or combinations of these compounds (Fig. 3a, b). PD0325901 dramatically inhibited T expression in the presence of CHIR99021 (Fig. 3a, Additional file 3: Figure S2A, B). The expression of T and FOXA2, which were upregulated by Activin A, was also prevented by PD0325901 (Fig. 3b, Additional file 3: Figure S2C, D). However, FGF2 only slightly induced T expression and could not induce FOXA2 expression (Fig. 3a, b), indicating that the activation of ERK alone may not efficiently induce EpiSC differentiation into the PS lineage. Real-time PCR showed that PD0325901 treatment significantly inhibited the expression of PS (*T*, *Mixl1*) and endoderm markers (*Foxa2*, *Sox17*) induced by Activin A and CHIR99021 (Fig. 3c, Additional file 3: Figure S2E, F). These results suggest that the basal ERK1/2 activity is essential for EpiSCs to differentiate into the PS, and PD0325901 promotes the commitment of neuroectodermal precursors through abrogating PS formation.

To investigate the role of ERK inhibition on self-renewal, mouse EpiSCs were treated with PD0325901, and SB431542 (a TGF-β pathway inhibitor), another inducer of neural lineage, was also tested. Consistent with a previous report [32], SB431542 inhibited NANOG expression and had little effect on OCT4 expression (Fig. 3d, e, Additional file 4: Figure S3A–D). However, PD0325901 treatment inhibited both NANOG and OCT4 efficiently, representing a more powerful effect for EpiSC differentiation (Fig. 3d, e, Additional file 4: Figure S3A–D). OCT4 is recognized as both a pluripotent protein and a PS inducer [13, 33]. Thus, decreased OCT4 expression by PD0325901 may inhibit both self-renewal and PS induction of EpiSCs. To test the possibility, we examined the effects of OCT4 on the expression of germ layer markers by knocking down *Oct4* with siRNA. Knockdown of *Oct4* decreased the expression of pluripotent markers (*Oct4*, *Nanog*) and increased the expression of PS markers (*T*, *Mixl1*) (Fig. 3f). These results suggested that PD0325901 treatment induces neuroectodermal precursors through prevention of primitive streak formation, as well as inhibiting the self-renewal of EpiSCs.

### ERK inhibition blocks primitive streak formation by modulating β-catenin localization

**Fig. 4:**
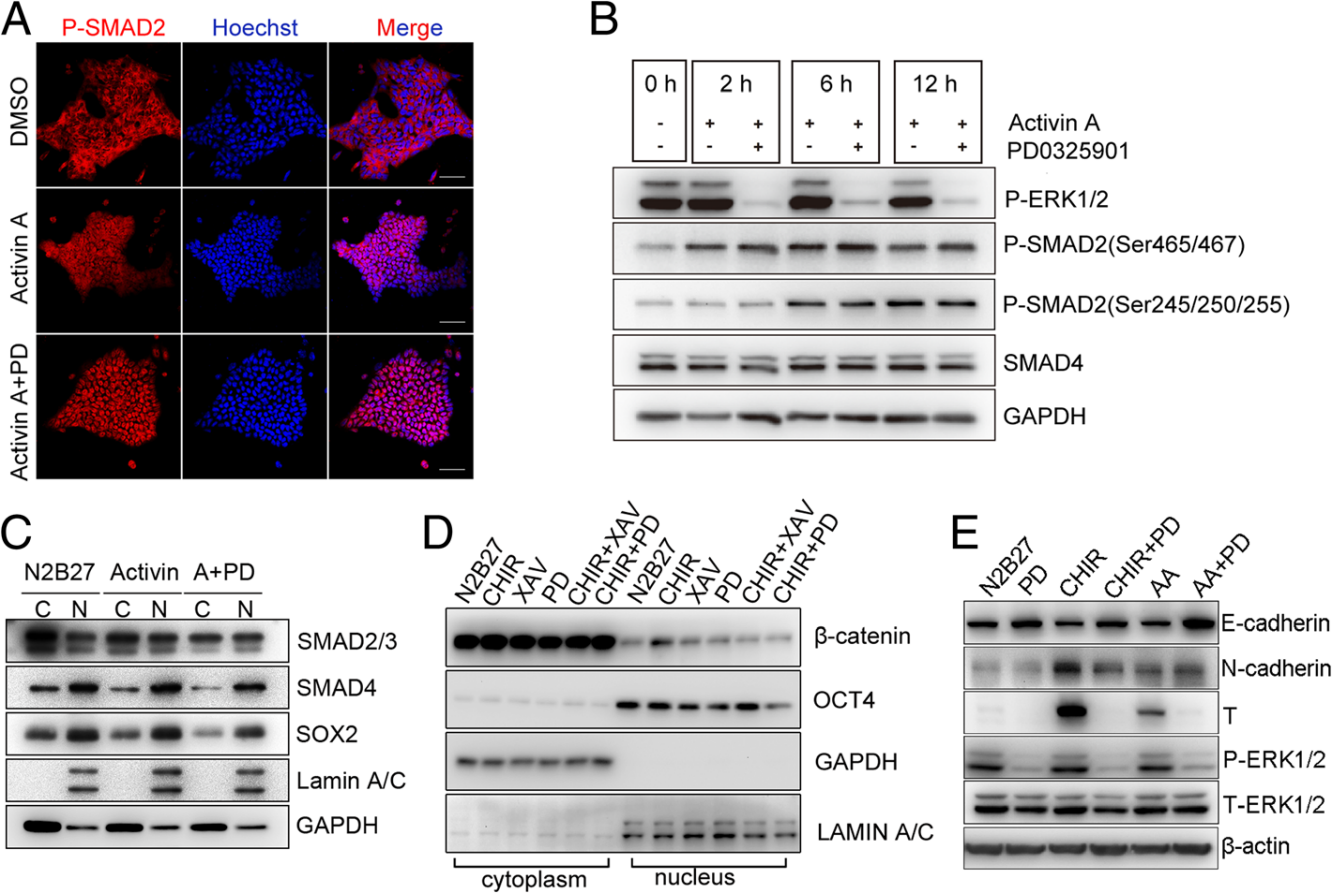
PD0325901 blocked formation of the primitive streak by preventing β-catenin accumulation in the nucleus. **a** Immunostaining showed that PD0325901 could not prevent the translocation of SMAD2 into nucleus in EpiSCs induced by Activin A. Bar, 100 μm. **b** Inhibition of phospho-ERK1/2 by PD0325901 did not affect phospho-SMAD2 and SMAD4 detected by western blot analysis. **c** Locations of SMAD2/3/4 were not changed in EpiSCs after PD0325901 treatment for 24 hours. C cytoplasm, N nucleus. **d** Western blot analysis showed that PD0325901 inhibited translocation of β-catenin into the nucleus. XAV939 (XAV, 2 μM) was used as a positive control that promoted the retention of β-catenin in cytoplasm. **e** PD0325901 increased expression of E-cadherin protein upon N2B27, CHIR99021, and Activin A treatment. SMAD SMAD family member, DMSO dimethyl sulfoxide, PD PD0325901, ERK extracellular signal-regulated protein kinase, SOX2 sex determining region Y-box 2, CHIR CHIR99021, OCT4 octamer-binding transcription factor 4, T brachyury

To explore how PD0325901 was involved in PS inhibition, we examined the effect of PD0325901 on the Activin A/SMAD and β-catenin pathways during EpiSC differentiation. Our results showed that SMAD2 translocated from cytoplasm to the nucleus after Activin A stimulation, but PD0325901 did not affect the location of SMAD2 (Fig. 4a). By the way, no significant difference was found on the levels of SMAD2 phosphorylation (at Ser465 and Ser467) after PD0325901 treatment (Fig. 4b, Additional file 5: Figure S4A). Although the linker region (at Ser245, Ser250, and Ser255) of SMAD2 have been shown to be potential ERK phosphorylation sites [34–36], phosphorylation of the linker region was not affected by PD0325901 in our system (Fig. 4b, Additional file 5: Figure S4B). In addition, SMAD4 protein levels (Fig. 4b, Additional file 5: Figure S4C), as well as the localization of SMAD3 or SMAD4, were similar in both control and PD0325901 treatment groups (Fig. 4b, c). Thus, the ERK pathway may interact with the SMAD pathway indirectly during epiblast differentiation into the primitive streak. The detailed mechanisms still need to be explored.

Then, we examined the levels of β-catenin in the cytoplasm and nucleus during the differentiation of EpiSCs. CHIR99021 treatment increased the level of β-catenin in the nucleus (Fig. 4d, Additional file 5: Figure S4D). However, PD0325901 treatment decreased the level of β-catenin in the nucleus as well as the level of OCT4 (Fig. 4d). We also examined the effect of PD0325901 on the expression of E-cadherin, which could recruit β-catenin to the inner side of cell membrane. We found that PD0325901 significantly increased the expression of E-cadherin even in the presence of CHIR99021 and Activin A (Fig. 4e, Additional file 5: Figure S4E, F). These data indicate that the ERK pathway may crosstalk with the WNT/β-catenin pathway through E-cadherin.

### Low FGF/ERK activity contributes to mouse neuroectoderm development

**Fig. 5:**
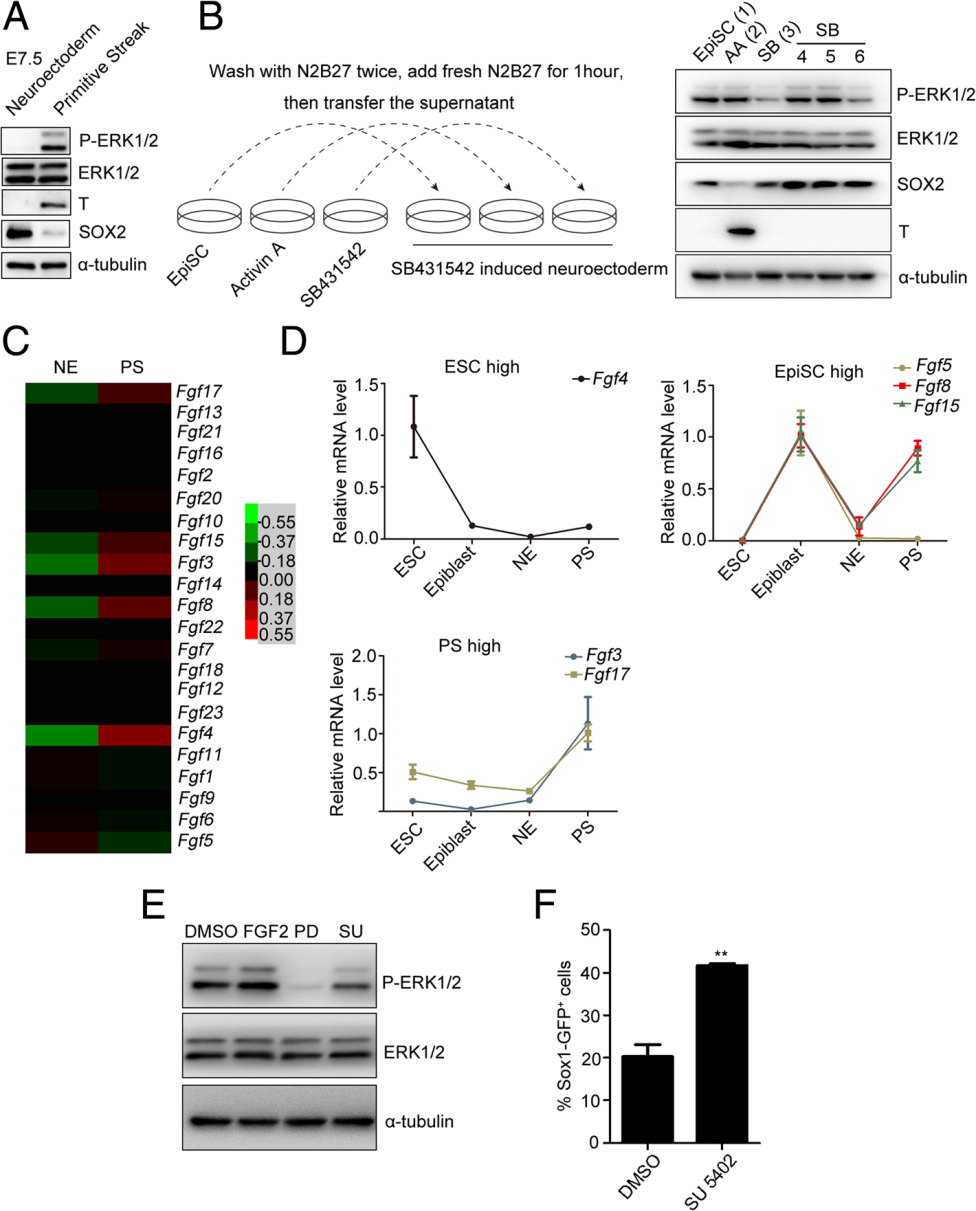
Low expression of FGF family genes accounted for the reduced ERK activity in the neuroectoderm. **a** Activity of ERK1/2 in the E7.5 neuroectoderm (NE) and primitive streak (PS) was detected by western blot analysis. **b** Secreted signals were responsible for regulation of ERK activity. Left panel shows manipulation of cells. Right panel shows western blot analysis results. Lanes 1–3 show EpiSCs, primitive streak (PS-like cells) induced by Activin A (AA) and neuroectoderm (NE-like cells) induced by SB431542 (SB). After 24 hours of differentiation, cells were washed with N2B27 twice, and refilled with fresh N2B27 medium for 1 hour. Supernatants were collected to stimulate NE-like cells. Lanes 4–6 show SB-induced NE-like cells stimulated for 1 hour with the supernatant from EpiSCs (lane 4), PS-like cells (lane 5), and NE-like cells (lane 6). **c** Heatmap showing relative mRNA levels of FGF family members detected by microarray in the NE and PS. Heatmap was generated by Cluster 3.0. **d** Expression of main *Fgf* family members in ESCs, E6.5 epiblasts, NE, and PS examined by real-time PCR. Expression patterns classified into three categories characterized by high expression in ESCs, epiblasts, and PS, respectively. **e** Phospho-ERK1/2 of EpiSCs detected by western blot analysis after treatment with DMSO, 5 ng/ml FGF2, 1 μM PD0325901, or 2 μM SU-5402 in N2B27 for 6 hours. **f** FACS showed that FGFR inhibitor SU-5402 treatment on Sox1-GFP EpiSCs increased the proportion of GFP-positive cells. **p < 0.01. AA Activin A, DMSO dimethyl sulfoxide, E embryonic day, EpiSC epiblast stem cell, ESC embryonic stem cell, ERK extracellular signal-regulated protein kinase, SOX2 sex determining region Y-box 2, T brachyury, FGF fibroblast growth factor, GFP green fluorescent protein

To seek in-vivo evidence for neuroectoderm differentiation, we separated the E7.5 epiblast into neuroectoderm (NE) and PS and examined the levels of phosphorylated ERK1/2, total ERK1/2, T, and SOX2 proteins in these tissues by western blot analysis. As anticipated, T was highly expressed in the PS, whereas SOX2 was expressed in the NE (Fig. 5a). Total ERK1/2 was highly expressed in both the PS and NE, while phospho-ERK1/2 in NE was much lower than that in PS (Fig. 5a). These data suggest that inhibition of ERK1/2 activity may be required for the generation of the NE during mouse embryonic development.

To examine whether extracellular signals were involved in the regulation of ERK activity, we stimulated NE-like cells with supernatants from PS-like cells, NE-like cells, or EpiSCs (Fig. 5b). Western blot analysis showed that NE-like cells had higher levels of phosphorylated ERK when they were stimulated with supernatant from PS-like cells compared to that from NE-like cells (Fig. 5b), implying that the different ERK responses were mainly from extracellular signals. In our previous study [37], we separated the neuroectoderm and primitive streak from E7.5 embryos, and performed microarray analysis. We screened the expression of *Fgf* family genes, well-known activators of ERK activity, in the neuroectoderm and primitive streak. Compared to the primitive streak, *Fgf* family members, including *Fgf3*, *Fgf4*, *Fgf8*, *Fgf15*, and *Fgf17*, were dramatically decreased in neuroectoderm (Fig. 5c, Additional file 1: Table S2). Real-time PCR showed that the expression patterns of *Fgf* family members could be classified into three groups: highly expressed in ESCs, epiblasts, and PS, respectively, with no *Fgf* member upregulated in the neuroectoderm (Fig. 5d). Therefore, these results suggest that the low expression of *Fgf* family members in the neuroectoderm may account for the low activity of ERK in neuroectoderm during mouse embryonic development.

To further investigate the regulation of ERK activation by FGF, the level of phospho-ERK1/2 during EpiSC differentiation was examined by adding FGF2 and SU-5402, a specific inhibitor of FGFR. FGF2 treatment increased the level of phospho-ERK1/2, whereas SU-5402 treatment significantly decreased the phosphorylation of ERK1/2 (Fig. 5e). Furthermore, SU-5402 treatment significantly increased the percentage of GFP-positive cells during differentiation of Sox1-GFP mEpiSCs into neural precursors (Fig. 5f). These results indicate that the decreased expression of FGF members is responsible for reduced ERK activity, thereby contributing to the specification of neuroectodermal precursors in mouse embryonic development.

### ERK inhibition at the epiblast-like stage accelerates mouse ESC differentiation into neuroectodermal precursors

**Fig. 6:**
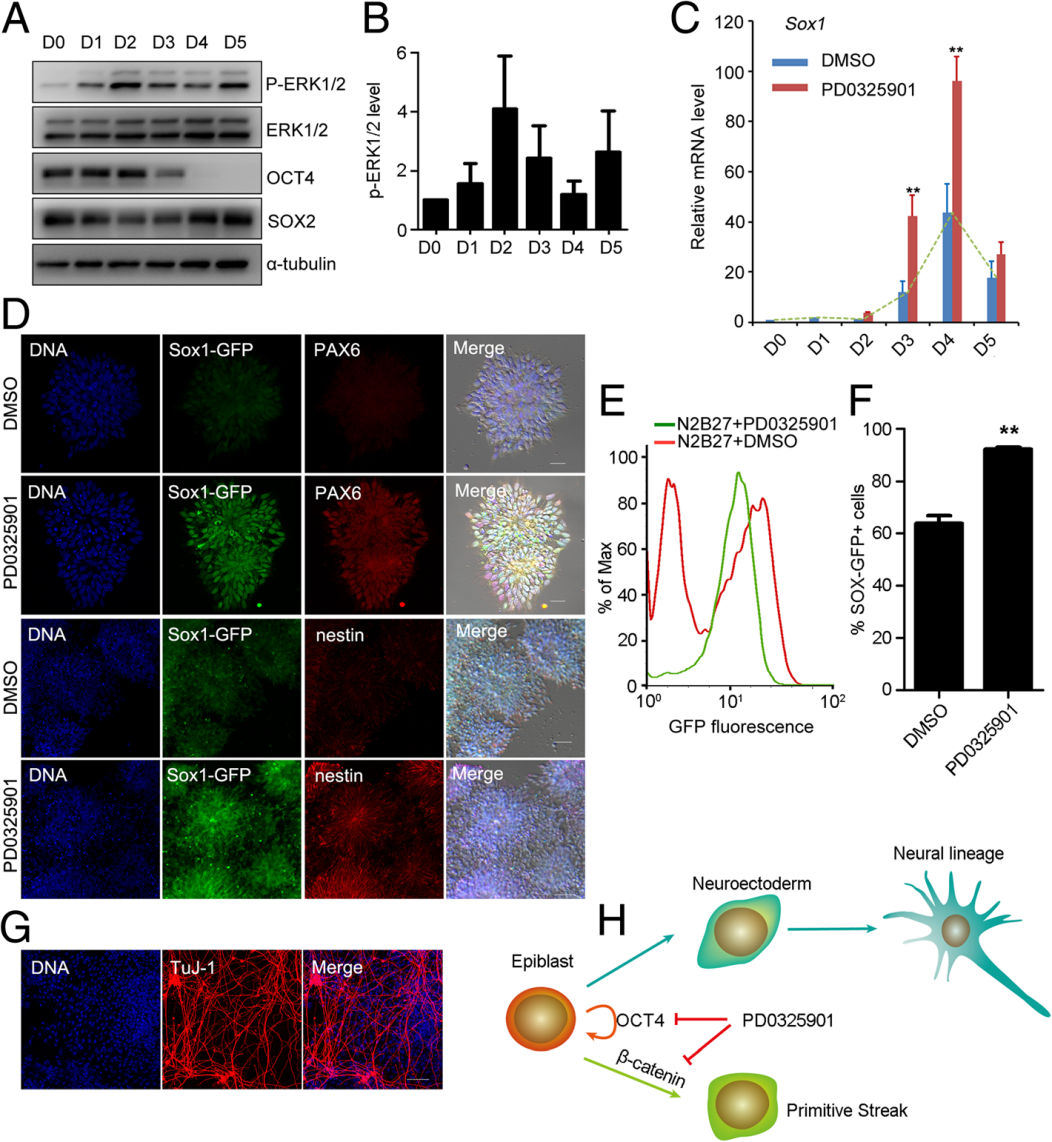
ERK inhibition at epiblast-like stage promoted differentiation of neuroectodermal precursors from ESCs. **a**, **b** ESCs cultured in N2B27 medium for neuroectodermal precursor commitment on gelatin-coated dishes. **a** Activity of ERK1/2 from D0 to D5 detected by western blot analysis. **b** Relative intensity of western blot analysis from (**a**) analyzed by ImageJ software. **c** PD0325901 treatment on day 3 (48–72 hours) or day 4 (72–96 hours) increased *Sox1* mRNA expression. **p < 0.01. **d** Immunostaining of neuroectoderm marker PAX6 and neural stem cell marker nestin. PD0325901 treatment (72–96 hours) increased expression of these markers. **e**, **f** FACS showed that PD0325901 treatment (72–96 hours) increased GFP-positive cells during 46C ESC neural differentiation. **p < 0.01. **g** GFP-positive cells from PD0325901 treatment (72–96 hours) were further cultured in N2B27 for 3 days and stained with TuJ-1 antibody for neuron detection. Bar, 100 μm. **h** Working model of PD0325901 on the commitment of epiblast into neuroectoderm precursor cells. PD0325901 inhibits the self-renewal by decreasing the expression of pluripotent genes, and blocks the formation of primitive streak by preventing the accumulation of β-catenin in the nucleus, thus promoting the differentiation of epiblast into neuroectoderm precursor cells. D day, DMSO dimethyl sulfoxide, ERK extracellular signal-regulated protein kinase, OCT4 octamer-binding transcription factor 4, *Sox1* sex determining region Y-box 1, SOX2 sex determining region Y-box 2, GFP green fluorescent protein, *Pax6* paired box 6

Based on the observations described above, we hypothesized that manipulating the activity of ERK by PD0325901 at the epiblast-like stage might promote the commitment of neuroectodermal precursors during mouse ESC differentiation. To test this, we used mouse ESCs (Sox1-GFP cell line, 46C) to investigate the activity of ERK1/2 during early neural lineage specification in a monolayer culture system [21]. Western blot showed that the phospho-ERK1/2 level was highest at day 2 differentiation, decreased at day 3, and then increased again at day 5 (Fig. 6a, b). These data suggested that ERK signaling had distinct roles at different stages during neural differentiation. We then treated the differentiating cells with PD0325901 for 24 hours at different time points from day 1 to day 5 during neural differentiation. PD0325901 treatment on day 3 (from 48 to 72 hours) or day 4 (from 72 to 96 hours) significantly increased the expression of the neuroectodermal precursor marker (*Sox1*) (Fig. 6c), and dramatically decreased the expression of PS markers (*T* and *Mixl1*) (Additional file 6: Figure S5A–D). Immunostaining showed neural rosettes were formed in both DMSO control and PD0325901 treatment (from 72 to 96 hours) (Fig. 6d). Compared with DMSO control, the expression of neuroectoderm markers PAX6 and SOX1 and neural stem cell marker nestin was significantly enhanced after PD0325901 treatment (Fig. 6d, Additional file 6: Figure S5E). Furthermore, we quantified the percentage of GFP-positive cells at day 5 through FACS. GFP-positive cells were 63.9% when ESCs were differentiated spontaneously in N2B27 medium. PD0325901 treatment (from 72 to 96 hours) significantly promoted the proportion of GFP-positive cells to 92.1% (Fig. 6e, f).

To confirm the neuron potential of Sox1-GFP-positive cells (PD0325901 treatment from 72 to 96 hours), these cells were sorted by FACS, cultured in N2B27 for 3 days further, and stained with a TuJ-1 specific antibody. The results showed that Sox1-GFP-positive cells differentiated into TuJ-1-positive neuron cells (Fig. 6 g). Above all, ERK inhibition at the epiblast-like stage significantly promotes the commitment of neuroectodermal precursors during ESC differentiation.

Taken together, our results reveal that neuroectoderm differentiation does not require extrinsic signals to activate ERK during epiblast differentiation. The inhibition of ERK1/2 decreases the expression of OCT4 and NANOG, blocking the self-renewal of EpiSCs. Meanwhile, primitive streak lineage differentiation is blocked by preventing the accumulation of β-catenin in the nucleus (Fig. 6 h). Thus, in the presence of PD0325901, EpiSCs differentiate into neuroectodermal lineage more efficiently.

## Discussion

Mouse ESCs can differentiate into EpiSC-like cells by culturing in EpiSC medium [24, 38, 39]. On the contrary, mouse EpiSCs can be reverted to ESCs by passaging in 2i/LIF or serum/LIF medium with low efficiency [24, 40]. In the present study, we showed that most epiblasts and EpiSCs differentiated into the neural lineage in 2i/LIF medium, in which PD0325901 was responsible for neural differentiation. In our system, we did not observe ESC clone formation from EpiSCs cultured in 2i/LIF, supporting the ultra-low reprogramming efficiency from epiblast to ESCs. Previously, Piliszek et al. [41] found that ERK inhibition abolishes primitive endoderm formation in rabbit embryos. Consistently, our study showed that ERK inhibition promoted neuroectodermal precursor commitment by blocking self-renewal and primitive streak formation of epiblast. These results suggest ERK signaling plays multiple roles in epiblast differentiation and self-renewal.

Hyperactive ERK inhibits SMAD-dependent TGF-β responses in the mouse mammary epithelial cell system through phosphorylation of SMAD2 at a cluster of serine–proline sites within its linker region, including Ser245, Ser250, and Ser255 [42]. On the contrary, elevated ERK activity enhances TGF-β-dependent responses in human mesangial cells [43]. Here, we found that PD0325901 efficiently prevented the expression of primitive streak markers induced by Activin A, suggesting potential crosstalk between ERK and the SMAD pathway. The linker domain of the SMAD family has been shown to be a potential ERK phosphorylation site [34–36]. However, we did not observe the effect of PD0325901 on phosphorylation of SMAD2 (at Ser245, Ser250, and Ser255) linker region (Fig. 4b). Furthermore, SMAD4 protein and SMAD2 phosphorylation (at Ser465 and Ser467) levels were comparable between control and PD0325901 treatment groups (Fig. 4b). In addition, PD0325901 treatment did not affect the localization of SMAD2, SMAD3, or SMAD4 (Fig. 4c). These results imply an indirect crosstalk between ERK and the SMAD pathway. In fact, primitive streak induction is regulated by β-catenin through collaborative interactions with SMAD2/SMAD3 and OCT4 [13]. Thus, PD0325901 may block Activin A-induced PS formation through β-catenin. In an *Fgfr1*-deficient mouse model, E-cadherin can bind and stabilize β-catenin at the membrane, which attenuates WNT signaling and prevents primitive streak formation [44]. Consistently, our results showed that PD0325901 treatment promoted the retention of β-catenin in cytoplasm. Meanwhile, E-cadherin was upregulated by PD0325901 in the presence of Activin A (Fig. 4d, e). These results suggest that PD0325901 may block Activin A-induced PS formation through E-cadherin/β-catenin.

## Conclusions

Our results suggest that inhibition of FGF/ERK signaling promotes EpiSC neuroectodermal lineage commitment through blocking EpiSC self-renewal and PS differentiation (Fig. 6 h). Based on our model, we provided a new strategy for efficient neural differentiation from EpiSCs and ESCs using a single inhibitor. Meanwhile, we observed decreased ERK activity in the neuroectoderm in E7.5 embryos. Thus, this strategy obeys the developmental rule, representing a more superior method for differentiation of ESCs into neuroectodermal lineage. Our research provides important insights into the role of FGF/ERK signaling on specification of the neural lineage during mouse early embryonic development, and contributes to the directed differentiation of stem cells in vitro and the clinical usage of stem cells for regenerative medicine.

## Additional files

Additional file 1: **Table S1** presenting primer sequences for real-time PCR and **Table S2** presenting expression patterns of *Fgf* family members in neuroectoderm and primitive streak. (DOC 61 kb)

Additional file 2: Figure S1. Showing quantitative analysis of western blot assay results related to Fig. 2a. Quantitative analysis of active β-catenin (**A**), p-ERK1/2 (**B**), and p-STAT3 (**C**) proteins performed using ImageJ software. *p < 0.05. (TIF 117 kb)

Additional file 3: Figure S2. Showing PD0325901 prevents formation of the PS. (**A**, **B**) Quantitative analysis of pERK1/2 and T proteins related to Fig. 3a. (**C**, **D**) Quantitative analysis of T and FOXA2 proteins related to Fig. 3b. (**E**, **F**) Real-time PCR showed PD0325901 decreased the differentiation of PS and endoderm in the presence of Activin A or CHIR99021 in EpiSCs cultured in N2B27 for 24 hours. Primitive streak marker, *Mixl1*; endoderm marker, *Sox17*. **p < 0.01. (TIF 216 kb)

Additional file 4: Figure S3. Showing quantitative analysis of immunofluorescence and western blot assay results related to Fig. 3d, e. (**A**, **C**) Quantitative analysis of OCT4 protein expression. (**B**, **D**) Quantitative analysis of NANOG protein expression. *p < 0.05, **p < 0.01. (TIF 132 kb)

Additional file 5: Figure S4. Showing quantitative analysis of western blot assay results related to Fig. 4b, d, e. (**A–C**) Quantitative analysis of p-SMAD2(Ser465/467), p-SMAD2(Ser245/250255), and SMAD4 in Fig. 4b. (**D**) Quantitative analysis of nuclear β-catenin protein in Fig. 4d. (**E**, **F**) Quantitative analysis of E-cadherin and N-cadherin proteins in Fig. 4e. *p < 0.05. (TIF 192 kb)

Additional file 6: Figure S5. Showing ERK inhibition at the epiblast-like stage inhibits the differentiation of PS and EpiSC self-renewal. (**A–D**) PD0325901 treatment for 24 hours at different time windows affected ESC commitment. PD0325901 treatment on day 2 increased *Nanog* expression. PD0325901 treatment on day 3 or 4 decreased expression of *Oct4*, *T*, and *Mixl1*. (**E**) Quantitative analysis of immunofluorescence results related to Fig. 6d. *p < 0.05 (TIF 288 kb)

## Abbreviations

BMP: Bone morphogenetic protein
CHIR: CHIR99021
EpiSC: Epiblast stem cell
ERK: Extracellular signal-regulated protein kinase
ESC: Embryonic stem cell
FGF: Fibroblast growth factor
*Fgfr*: Fibroblast growth factor receptor
FOXA2: Forkhead box protein A2
*Gapdh*: Glyceraldehyde-3-phosphate dehydrogenase
GFP: Green fluorescent protein
*Hprt*: Hypoxanthine-guanine phosophoribosyltransferase
LIF: Leukemia inhibitory factor
MEF: Mouse embryonic fibroblasts
*Mixl1*: Mix paired-like homeobox
NE: Neuroectoderm
Nes-Cre: Nestin-Cre
OCT4: Octamer-binding transcription factor 4
*Pax6*: Paired box 6
PD: PD0325901
PS: Primitive streak
SMAD2: SMAD family member 2
*Sox1*: Sex determining region Y-box 1
SOX2: Sex determining region Y-box 2
STAT3: Signal transducer and activator of transcription 3
T: Brachyury
TuJ-1: Neuron-specific class III beta-tubulin
WNT: Wingless-type MMTV integration site family, member
