# Supplementary material for: ERK inhibition promotes neuroectodermal precursor commitment by blocking self-renewal and primitive streak formation of the epiblast
Source: Stem Cell Res Ther. 2018 Jan 5;9:2. doi: 10.1186/s13287-017-0750-8 (PMC5756365; doi:10.1186/s13287-017-0750-8)
Supplement: Supplementary file 1 — Table S1 presenting primer sequences for real-time PCR and Table S2 presenting expression patterns of Fgf family members in neuroectoderm and primitive streak. (DOC 61 kb) [file 13287_2017_750_MOESM1_ESM.doc]

**Supplemental tables**

**ERK inhibition promotes neuroectodermal precursor commitment by blocking self-renewal and primitive streak formation of epiblast**

Yang Yu^1,2^, Xiaoxiao Wang^1,2^, Xiaoxin Zhang^1^, Yanhua Zhai^1^, Xukun Lu^1,2^, Haixia Ma^1,2^, Kai Zhu^1,2^, Tongbiao Zhao^1^, Jianwei Jiao^1^, Zhen-Ao Zhao^1, 3,*^, Lei Li^1,*^

**Supplemental tables**

**Table S1 Primer sequences for real-time PCR**

| **Gene** | **Forward sequences** | Reverse **sequences** |
| --- | --- | --- |
| *Oct4* | GAAGCAGAAGAGGATCACCTTG | TTCTTAAGGCTGAGCTGCAAG |
| *Nanog* | TGCTCCGCTCCATAACTTCG | GGCTTGTGGGGTGCTAAAAT |
| *Sox1* | AGACTTCGAGCCGACAAGAG | AACTGTGCAAACAGGTGCAG |
| *Sox2* | GGCAGCTACAGCATGATGCAGGAGC | CTGGTCATGGAGTTGTACTGCAGG |
| *Pax6* | TAACGGAGAAGACTCGGATGAAGC | CGGGCAAACACATCTGGATAATGG |
| *nestin* | AGGCTGAGAACTCTCGCTTGC | GGTGCTGGTCCTCTGGTATCC |
| *T* | CATCGGAACAGCTCTCCAACCTAT | GTGGGCTGGCGTTATGACTCA |
| *Mixl1* | CGCTCCCTCAGTAACAACGC | GCTGCCACAGACTTCCAAATG |
| *Sox17* | CTTTATGGTGTGGGCCAAAG | TTCCAAGACTTGCCTAGCATC |
| *Foxa2* | CCATCAGCCCCACAAAATG | CCAAGCTGCCTGGCATG |
| *Fgf3* | GCCGCGCCTTCGTGAGA | AAGCCAGATCAGGCCCATCC |
| *Fgf4* | GGTGGCTCACAGGACAATAAGA | GCCTGGGCTATGAGACCGT |
| *Fgf5* | GAAATATTTGCTGTGTCTCAGGG | TAAATTTGGCACTTGCATGG |
| *Fgf8* | GTGGAGACCGATACTTTTGGAA | CCTTGCCTTTGCCGTTGC |
| *Fgf15* | GACTGCGAGGAGGACCAAAA | CAGCCCGTATATCTTGCCGT |
| *Fgf17* | CAGTAGCCCAAGAGAGCGAG | TCTCGGAGCCACAGGTTTTC |
| *Hprt* | GCCAGTAAAATTAGCAGGTGTTCT | ATAGGCTCATAGTGCAAATCAAAAG |

**Table S2 Expression patterns of Fgf family members in neuroectoderm and primitive streak**

| **Gene symbol** | **neuroectoderm** | **primitive streak** |
| --- | --- | --- |
| *Fgf1* | 12.39697 | 10.1102 |
| *Fgf10* | 22.81723 | 26.59272 |
| *Fgf11* | 17.98748 | 14.62775 |
| *Fgf12* | 10.39676 | 11.66462 |
| *Fgf13* | 468.1753 | 493.3225 |
| *Fgf14* | 12.4268 | 13.25021 |
| *Fgf15* | 89.89168 | 624.6199 |
| *Fgf16* | 10.8194 | 11.96201 |
| *Fgf17* | 31.13911 | 131.7604 |
| *Fgf18* | 14.06975 | 15.80857 |
| *Fgf2* | 5.756841 | 5.805803 |
| *Fgf20* | 12.46585 | 14.91568 |
| *Fgf21* | 19.70505 | 20.31696 |
| *Fgf22* | 38.33007 | 40.69619 |
| *Fgf23* | 8.624135 | 8.745837 |
| *Fgf3* | 17.3634 | 177.4506 |
| *Fgf4* | 16.8396 | 325.7454 |
| *Fgf5* | 161.8955 | 56.09611 |
| *Fgf6* | 11.24955 | 9.973835 |
| *Fgf7* | 18.93204 | 26.8522 |
| *Fgf8* | 39.87478 | 431.0339 |
| *Fgf9* | 75.00623 | 69.29653 |

mRNAs from neuroectoderm and primitive streak were detected by Affymetrix GeneChip® Mouse Genome 430 2.0 Array related to Figure 5.
