# Supplementary figures and images for: ERK inhibition promotes neuroectodermal precursor commitment by blocking self-renewal and primitive streak formation of the epiblast
Source: Stem Cell Res Ther. 2018 Jan 5;9:2. doi: 10.1186/s13287-017-0750-8 (PMC5756365; doi:10.1186/s13287-017-0750-8)

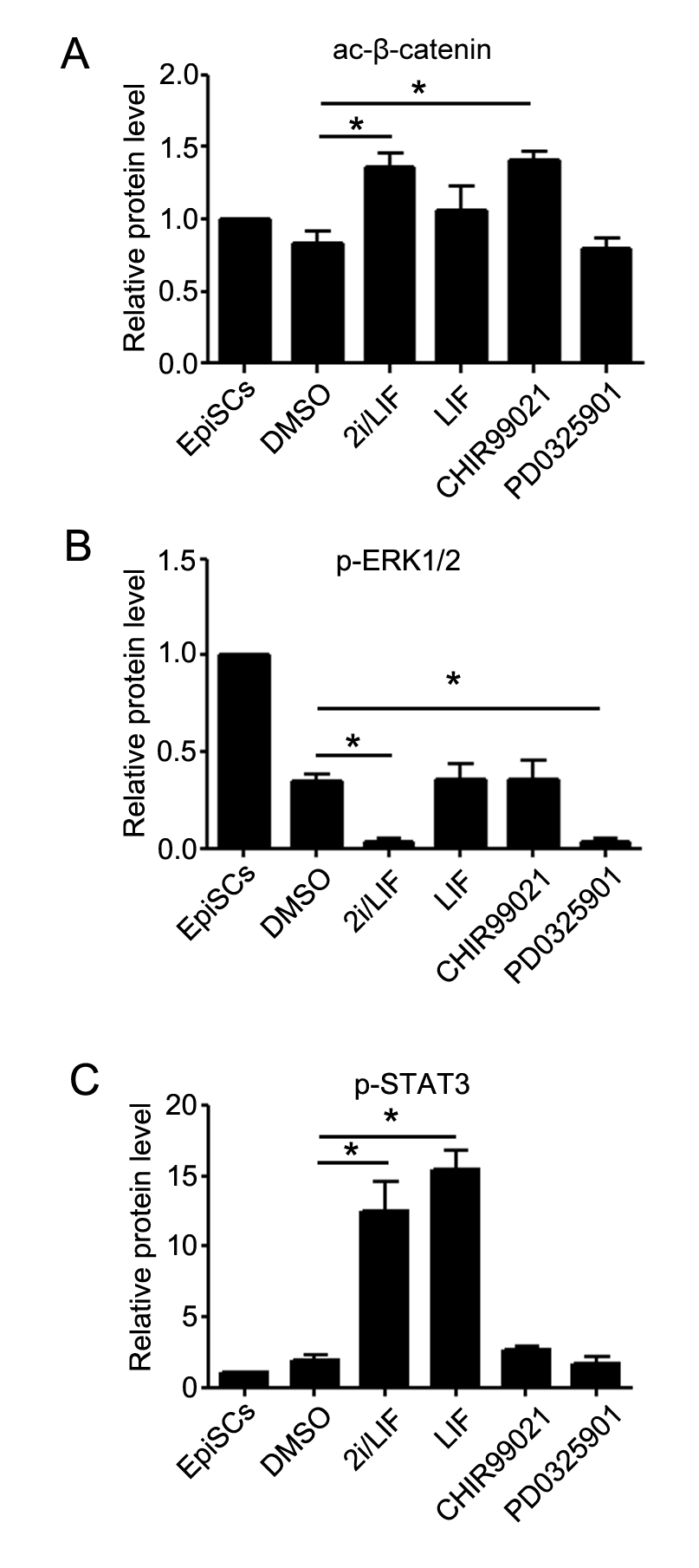

Supplement: Supplementary file 2 — Showing quantitative analysis of western blot assay results related to Fig. 2a. Quantitative analysis of active β-catenin (A), p-ERK1/2 (B), and p-STAT3 (C) proteins performed using ImageJ software. *p < 0.05. (TIF 117 kb) [file 13287_2017_750_MOESM2_ESM.tif]

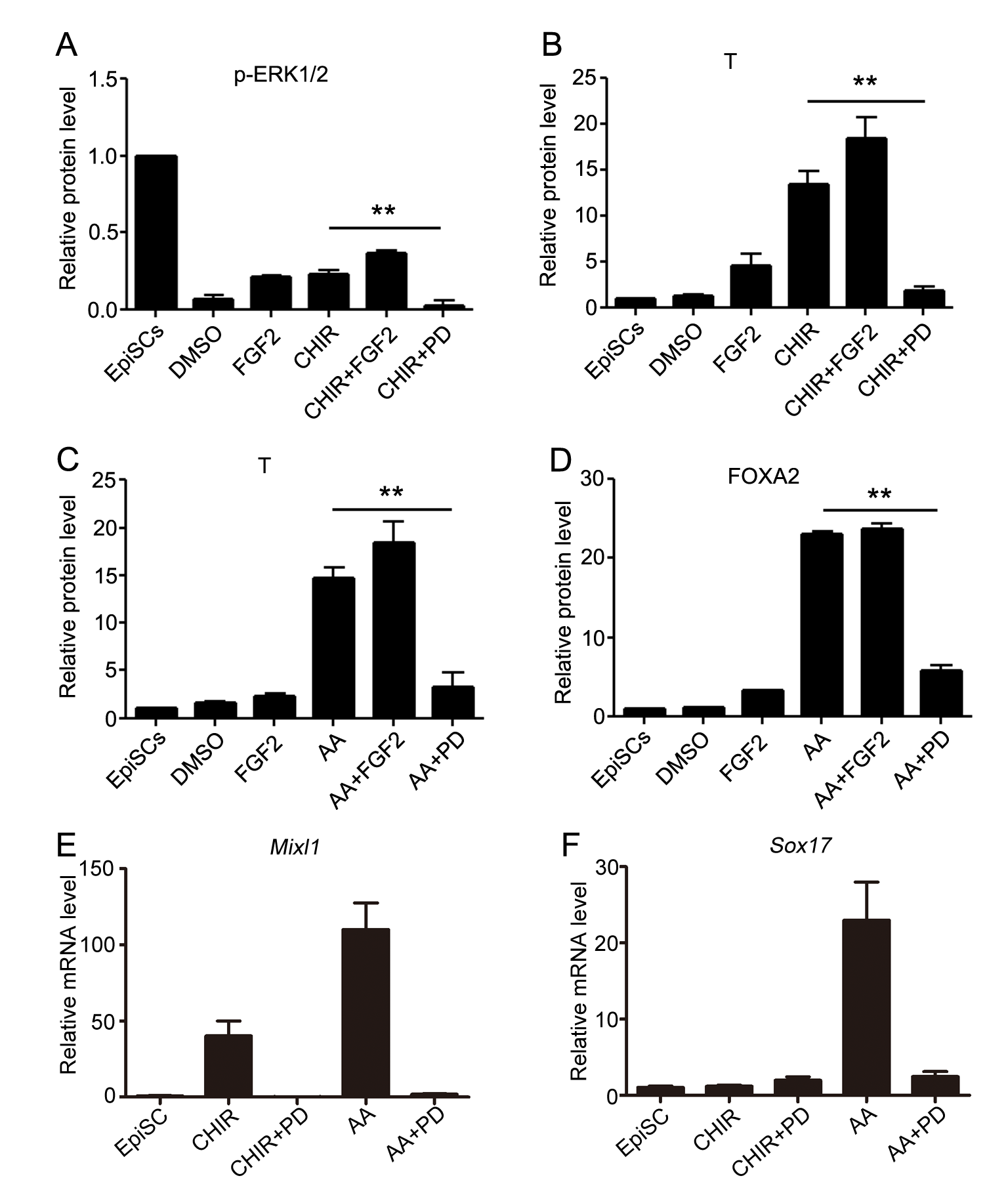

Supplement: Supplementary file 3 — Showing PD0325901 prevents formation of the PS. (A, B) Quantitative analysis of pERK1/2 and T proteins related to Fig. 3a. (C, D) Quantitative analysis of T and FOXA2 proteins related to Fig. 3b. (E, F) Real-time PCR showed PD0325901 decreased the differentiation of PS and endoderm in the presence of Activin A or CHIR99021 in EpiSCs cultured in N2B27 for 24 hours. Primitive streak marker, Mixl1; endoderm marker, Sox17. **p < 0.01. (TIF 216 kb) [file 13287_2017_750_MOESM3_ESM.tif]

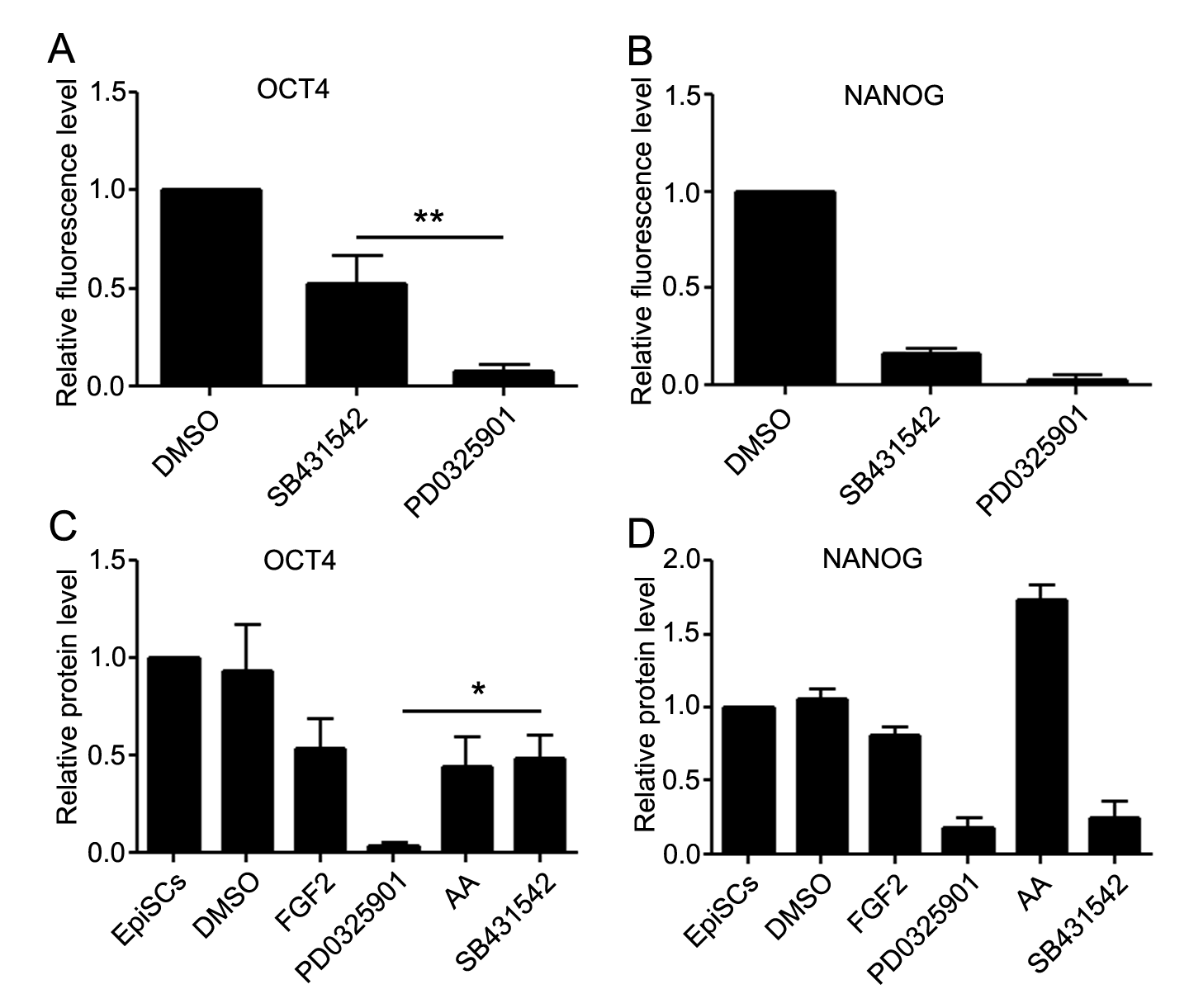

Supplement: Supplementary file 4 — Showing quantitative analysis of immunofluorescence and western blot assay results related to Fig. 3d, e. (A, C) Quantitative analysis of OCT4 protein expression. (B, D) Quantitative analysis of NANOG protein expression. *p < 0.05, **p < 0.01. (TIF 132 kb) [file 13287_2017_750_MOESM4_ESM.tif]

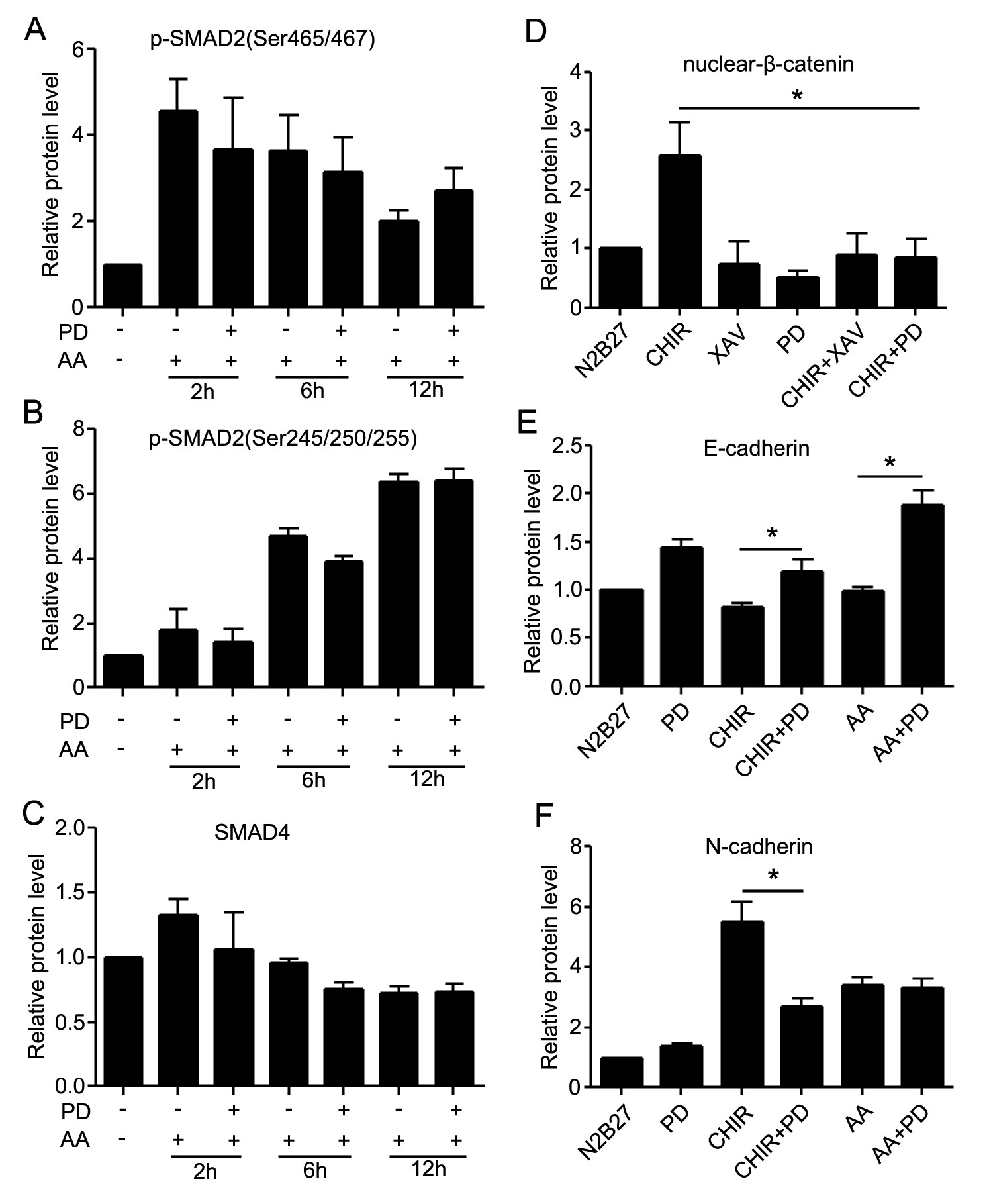

Supplement: Supplementary file 5 — Showing quantitative analysis of western blot assay results related to Fig. 4b, d, e. (A–C) Quantitative analysis of p-SMAD2(Ser465/467), p-SMAD2(Ser245/250255), and SMAD4 in Fig. 4b. (D) Quantitative analysis of nuclear β-catenin protein in Fig. 4d. (E, F) Quantitative analysis of E-cadherin and N-cadherin proteins in Fig. 4e. *p < 0.05. (TIF 192 kb) [file 13287_2017_750_MOESM5_ESM.tif]

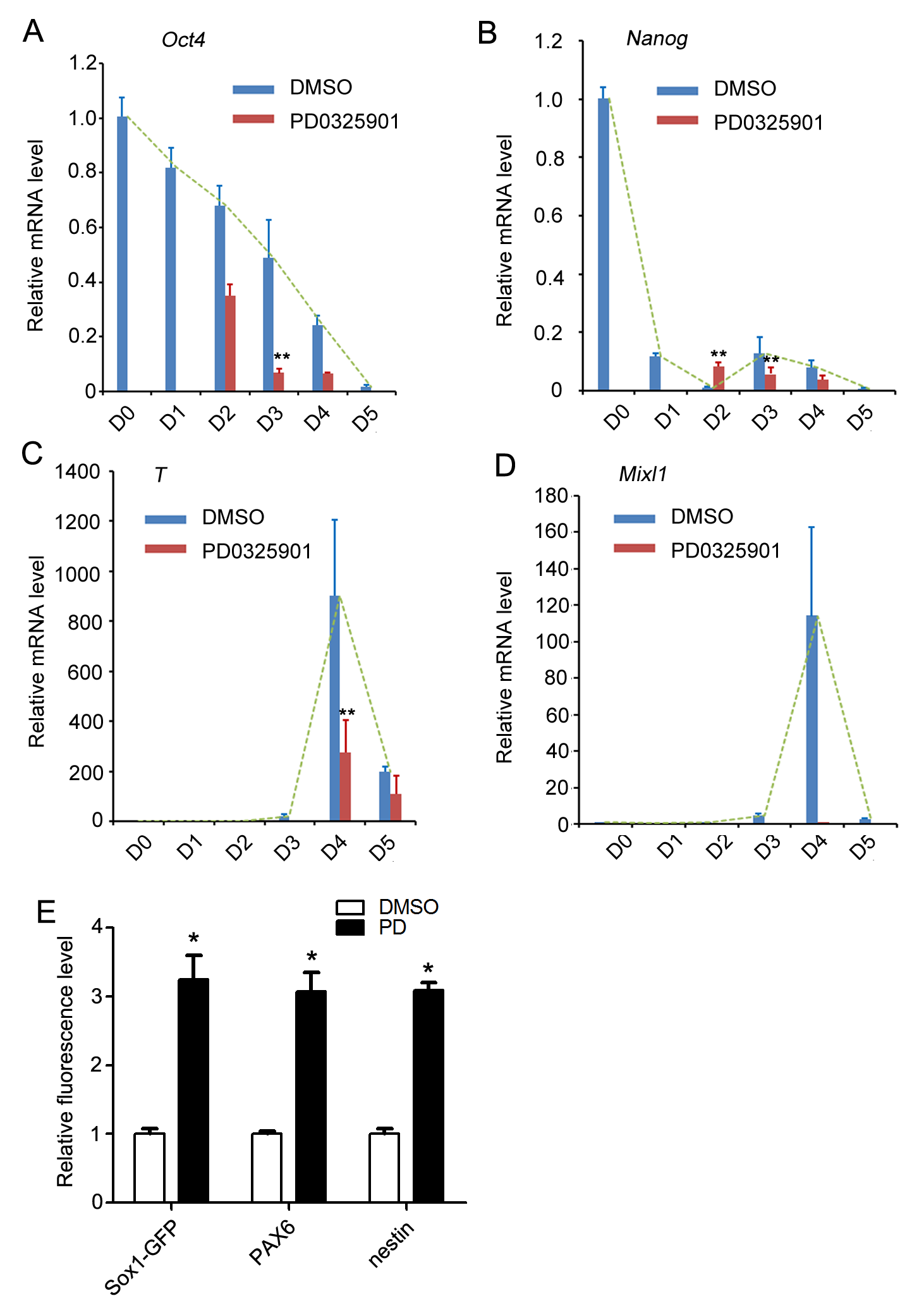

Supplement: Supplementary file 6 — Showing ERK inhibition at the epiblast-like stage inhibits the differentiation of PS and EpiSC self-renewal. (A–D) PD0325901 treatment for 24 hours at different time windows affected ESC commitment. PD0325901 treatment on day 2 increased Nanog expression. PD0325901 treatment on day 3 or 4 decreased expression of Oct4, T, and Mixl1. (E) Quantitative analysis of immunofluorescence results related to Fig. 6d. *p < 0.05 (TIF 288 kb) [file 13287_2017_750_MOESM6_ESM.tif]
